# More Talent, More Leeway: Do Violence Against Women Arrests Really Hurt NFL Player Careers?

**DOI:** 10.1177/10778012221092477

**Published:** 2022-05-06

**Authors:** Daniel Sailofsky

**Affiliations:** 1 4907 Middlesex University London, The Burroughs, Hendon, London, UK

**Keywords:** conflict theory, matched pairs, NFL, deterrence, violence against women

## Abstract

This article examines whether arrests for an act of violence against women have a negative impact on National Football League (NFL) player careers and whether this impact has become more negative over time. Framed by criminological deterrence and conflict theories, I conduct a Bayesian multi-level negative binomial regression on a matched pairs sample of all 117 NFL players arrested for an act of violence against women between 2000 and 2019 (*n* = 234). Results show that the effect of an arrest on player careers is negligible, though it has become slightly more detrimental over time. Player value and performance are stronger predictors of post-arrest career trajectories, and average or better performance negates any detrimental impact of an arrest.

## Introduction

National Football League (NFL) player arrests are a common occurrence, with over 1,000 arrests recorded since 2000^1^ (Schrotenboer, 2021). While many of these arrests are for non-violent crimes, arrests for acts of violence against women (hereafter “VAW”) are a serious problem among NFL players (Leal et al., 2015; Withers, 2010), one which the NFL has historically not sufficiently addressed. Several studies have found that athletes in general (Leal et al., 2015; Young et al., 2017) and football players specifically (Gage, 2008; Leal et al., 2015) commit and are arrested for more acts of VAW than men in the general population, though these findings are not unanimous (McCray, 2015; Schwartz, 2021). While research suggests that sanctions for athlete acts of VAW are negligible (Sailofsky & Shor, 2022; Withers, 2010), many sports fans still believe that arrests and criminal allegations are likely to ruin an athlete's career (Delgado, 2014; TMZ, 2018).

There is very little empirical work examining whether NFL players do indeed suffer either informal or formal sanctions due to arrests for acts of VAW.^2^ While scholars have examined the specific one-time sanctions (usually fines and suspensions) given to professional athletes involved in violent acts (Brown, 2016), there has been no comprehensive examination of the career outcomes of NFL players following arrests for acts of VAW. It therefore remains unclear whether these arrests affect the careers of professional football players, or how the effect of VAW arrests have changed over time.

If sports leagues hope to curb violent off-the-field behaviors by athletes, deterrence perspectives suggest that players should indeed pay a clear price for their actions in the form of tangible sanctions related to career prospects. Such sanctions might deter future acts and serve as a warning sign for other athletes, while also helping to set and reinforce norms of acceptable behavior in society more generally (Pickett & Baker, 2017, p. 218). From a criminological conflict theory perspective, the answer to the question of whether NFL players will be sanctioned is not straightforward. On one hand, NFL players disproportionately belong to a traditionally over-criminalized racial group (Davenport et al., 2011; Vuolo et al., 2017), suggesting that they may be sanctioned more heavily following acts of violence. The majority of NFL players are Black (Lawrence, 2019; Tapp, 2014), and Black men in the United States are arrested more often, charged more frequently and with harsher crimes, and imprisoned at higher rates than other racial groups (Latzer, 2018; Monk, 2019). This over-criminalization is particularly relevant in situations of violent crime, and as such, we might expect harsher sanctions for this type of alleged criminal behavior by Black athletes (Christensen et al., 2016). As bell hooks wrote, “Black males today live in a world that pays them the most attention when they are violently acting out” (2004, p. 53).

On the other hand, NFL players have also recently joined a traditionally under-criminalized and under-sanctioned economic class, one which is often able to buy its way out of criminal charges or avoid them altogether (Lanier, 2018; Reiman, 2015). As football players living in the United States, these athletes have often received preferential treatment from coaches, college athletic departments, and sometimes the justice system throughout their athletic careers (Beaver, 2019; Kreager, 2007). NFL players also have the backing of large corporate enterprises who have a financial incentive to minimize negative news and incidents regarding player behavior (Thomas, 2017). This study's questions therefore sit at an interesting nexus between gender, race, class, and social status.

Beyond determining whether NFL players arrested for acts of VAW in the past 20 years have suffered career consequences, the current study examines whether these consequences have changed over time. Awareness around the prevalence of VAW by NFL players has grown over the past 20 years (Bruton et al., 2018), which could impact the effect of these arrests on career outcomes. More specifically, the highly public 2014 domestic violence incident involving Baltimore Ravens running back Ray Rice could be an important “tipping point” moment for how NFL decision-makers handle players accused of acts of VAW (Chase, 2019).

To examine the effects of arrests following VAW on NFL player career outcomes, I collected data on all the NFL players who were arrested on such charges between the years 2000 and 2019 (*n* = 117). I matched each of these players with a player who was not arrested but was as similar as possible in terms of their age, race, position, draft position, career achievements, and relevant player statistics. Examining the career duration of these players in subsequent years thus allowed for the examination of the common notion that players pay a substantial price merely for being accused of a violent act and arrested for it, even if they are not eventually convicted.

## Research on VAW and Deviance By Athletes

While data on domestic and sexual violence complaints can be difficult to compile and acts of VAW are often underreported (Fugate et al., 2005), researchers have attempted to determine the prevalence of these incidents by professional athletes. Leal et al. (2015) found that between 2000 and 2013, there were 199 arrests for violent crime among NFL players, and found that NFL players had a higher violent crime rate than those in the general population over this period, though not a higher crime rate in general. Similarly, Leal et al. (2019) found that NFL players who have been arrested before they enter the NFL are more likely to be arrested for a violent crime while in the NFL, though not more likely to be arrested in general or for a non-violent crime. Research on NFL criminality and deviance has often relied on Schrotenboer's (2021) *USA Today* database, which tracks all player arrests since the year 2000.

In terms of NFL response to athlete-perpetrated VAW, researchers have often examined how league policies govern allegations and convictions for these incidents. While professional sports leagues and teams do pay some attention to VAW, they do not legislate off-field VAW as often and as swiftly as they do on-field violence (Brown, 2016; Lott, 2019). Moreover, throughout the NFL's history, punishments (in the form of suspensions and fines) for violent acts have been less severe than those for drug-related offenses (McCann, 2014).

Most of the literature surrounding professional athlete behavior and official league sanctions has also looked at the NFL, where arrests and convictions for acts of VAW have been most common among all major professional leagues in the United States (Brown, 2016; Leal et al., 2015). However, previous studies have often looked at the disciplinary authority and powers of commissioners, leagues, and teams (Lott, 2019) following acts of VAW, and did not account for the effects of these acts on athletes’ careers. Suspensions, fines, or mandated counseling have a one-time, short-term impact, and these studies therefore could not capture the effect that arrests following VAW might have on the accused athlete’s entire career and earning potential.

## The NFL'S Personal Conduct Policy (PCP) and the Ray Rice Incident

The NFL's policy surrounding acts of VAW is embedded in its PCP and has been altered twice since 2000. The first change, in 2007, was brought about by a sharp increase in NFL player arrests in 2006. Weiss (2008) notes that this increase may have been due to changes made to the NFL's collective bargaining agreement (CBA) in 2006 when the CBA was altered to “more robustly protect players’ bonuses from forfeiture due to their off-field behavior” (Weiss, 2008, p. 307). The findings from Weiss’s study provide more evidence that punishment or loss of rewards may have a deterrent effect on NFL players, as in this case, removing a deterrent (the detrimental impact that negative off-field behavior could have on player salary bonuses) led to increased player arrests. In 2007, the commissioner of the NFL, Roger Goodell, updated the PCP to include harsher consequences for players guilty of conduct deemed detrimental to the league, even if they had not been convicted of any crime (Lott, 2019). This updated PCP was not specific to incidents of VAW, though these acts could of course fall under “detrimental conduct.”

It is impossible to discuss the NFL's PCP and VAW without mentioning the 2014 domestic violence case of Ray Rice, which has become “a discrete moment in time, one that divided sports history into pre- and post-Ray Rice categories” (Chase, 2019, pp. 93-94). On February 14, 2014, Rice, an All-Star running back for the Baltimore Ravens, was accused of domestic violence for allegedly striking his fiancée in the elevator of an Atlantic City Hotel (Van Natta Jr. & Valkenburg, 2014). On February 19th, TMZ released a video of Rice dragging his seemingly unconscious fiancée out of the elevator (Van Natta Jr. & Valkenburg, 2014). This changed the media and outside perception of the incident, and on March 27th, a grand jury indicted Rice on a felony aggravated assault charge, with a potential prison sentence of 3 to 5 years (Lott, 2019). After investigations from the NFL and commissioner’s office, Rice was subsequently suspended (without pay) for the first two games of the next NFL season (Van Natta Jr. & Valkenburg, 2014). At this point, many already found this to be a lenient punishment on the part of the NFL and Goodell. Amid backlash from the media, women’s rights and domestic violence prevention groups, and some players, Goodell admitted that the two-game suspension was too lenient, and promised to update the NFL’s PCP (Van Natta Jr. & Valkenburg, 2014).

On September 8th, after the first week of the NFL season, TMZ released a second video from inside the elevator, showing Rice knocking out his fiancée (TMZ, 2014). After this video was made public, Rice was released by the Ravens and indefinitely suspended by the NFL (Lott, 2019). Both the Ravens and Goodell denied having seen this video when they sanctioned Rice, even though both parties claimed to have completed thorough investigations.

The NFL's current PCP stems from this incident. This policy maintains the vague language of its predecessor, providing that “[e]veryone who is part of the league must refrain from 'conduct detrimental to the integrity of and public confidence in’ the NFL” (National Football League, 2014).

VAW falls explicitly under the NFL’s list of prohibited conduct, providing for “a special form of discipline that applies specifically to violations regarding assault, battery, domestic violence, dating violence, child abuse and other forms of family violence, or sexual assault” (Lott, 2019). This calls for a first-time offender to receive a minimum six-game suspension, with a second offense resulting in permanent expulsion from the league. While this updated PCP may seem to be a strong response from the NFL, its application has been uneven (Chase, 2019; Lott, 2019). Lott (2019) notes that the PCP gives “wide latitude” to the NFL, providing “the impression that the NFL is not taking a strong stance on combating domestic violence” (Lott, 2019, p. 135).

## Theoretical Framework

### Deterrence and Expressive Theories of Punishment

Criminological deterrence theory (Lanier et al., 2015) provides a useful framework for examining the persistence of violent acts by NFL players. Stemming from classical theories of crime, deterrence theorists posit that in order to deter crime, punishment must be sufficiently severe, certain, and swift (Lanier et al., 2015, Chapter 3). If the likely consequences or punishment for committing a particular crime outweigh its benefits, people will be discouraged from engaging in such behavior. Initially focused on formal legal punishment, deterrence theorists have expanded the definition of “consequences” or “punishment” to include legal and extralegal punishment and sanctions, including loss or anticipated loss of rewards, reputation, or prestige (Nagin, 2013). As such, an athlete's risk of losing earnings or employment opportunities may serve as an extralegal sanction that could have a similar deterring effect. These types of extralegal sanctions have been observed in non-sports contexts, as research has shown that individuals with prior convictions are less likely to receive callbacks for jobs (Agan & Starr, 2016). Importantly, this effect was even more pronounced for Black men (Agan, 2017; Vuolo et al., 2017), who make up a significant percentage of NFL players.

If NFL players did suffer negative career consequences, the deterrent effect of this sanctioning could extend beyond these specific players and even beyond the NFL or professional athlete contexts. Drawing from the early work of Emile Durkheim on the boundary-making function of punishment, expressive theories of punishment posit that sanctions for criminal or deviant behavior can also have broader educative-moralizing effects on the members of a society (Garland, 1993; Pickett & Baker, 2017). What a particular society deems as criminal—coupled with the certainty, severity, and swiftness of punishment for engaging in this criminal behavior—creates and sustains the social and moral norms of a society (Durkheim, 2014; Nagin, 2013). If certain criminal behavior is *not* punished, especially when it is committed publicly, “norms are weakened and shown to be less universal in their binding force” (Garland, 1993, p. 33).

While deterrence theory provides a framework for understanding the importance of punishment in deterring future incidents of VAW, NFL teams’ actual commitment to deterring such incidents cannot be taken for granted. While one can assume NFL teams *hope* that players are not accused of acts of VAW (given near-universal societal condemnation of such behavior), it is not clear how seriously teams take these accusations when assessing whether to retain or acquire an accused player. Unlike in general crime deterrence situations, where it is only those who commit the crime (and their family and friends) who are negatively impacted by the decision to punish, in this situation both the accused (the player) and the sanctioning body (the team) can be negatively impacted by the team’s decision to punish. If an NFL team punishes a talented, valuable player by releasing them from the team or by not signing them to a subsequent contract, both the team and the player likely suffer. For teams, the desire to retain or acquire a talented, productive, and/or valuable player accused of an act of VAW may supersede their desire to release the player and help deter future incidents of VAW. While the link between more certain and harsher punishment for acts of VAW and deterrence of future acts of the same nature may be clear, it is less clear whether teams will actually punish these players in the first place.

### Conflict Theories

Criminological conflict theories and theories of Black masculinity highlight potentially contrasting results to the question of whether NFL players are likely to suffer negative career consequences when accused of an act of VAW. While most NFL players belong to a traditionally over-criminalized and highly sanctioned racial group (Vuolo et al., 2017), conflict theorists also suggest that groups with high status and economic means have the power to determine what is criminal or deviant (Lanier, 2018) within a social and criminal system that benefits those with more social power and resources (Barkley, 2005). The fact that the majority of NFL players are both Black and wealthy thus presents an interesting theoretical puzzle when examining the consequences of their actions.

Academic literature is littered with studies demonstrating the unequal treatment of Black men in legal and criminal justice contexts (Davenport et al., 2011; D. Jacobs et al., 2007; D. Jacobs & Carmichael, 2001; Krivo et al., 2009). This includes work examining the differential policing of Black and White people (Calderon, 2000; Davenport et al., 2011; Sewell & Jefferson, 2016) and use of force in police encounters (Mears et al., 2017), the effects of neighborhood segregation on rates of violent crime (Krivo et al., 2009) and adverse health outcomes (Sewell, 2015), and the higher imprisonment rates of Black men (Alexander, 2020; Keen & Jacobs, 2009; Kilgore, 2015), especially in states with higher levels of perceived racial threat (D. Jacobs & Carmichael, 2001). Similar unequal treatment has been observed in employment (Pager, 2003, 2008; Western & Pettit, 2005) following contact with the criminal justice system. Black men are less likely than White men to receive call-backs for jobs when they have a felony conviction on their record (Pager, 2003, 2008; Vuolo et al., 2017), and they are paid lower wages when they are hired (Western & Pettit, 2005). Black men also receive fewer call-backs when they are arrested for more minor crimes, even when they are not convicted (Uggen et al., 2014).

It is also important to highlight the treatment and framing of Black athletes and Black masculinity (Collins, 1990; hooks, 2004a, 2004b) throughout sports history (Carrington, 1998). The success of Black males in violent, contact-based sports like football has been used to “reinforce the fixed idea that Black men are ‘all brawn and no brains’” (Mercer, 2013, p. 178). In the sport context, Black men's bodies are often positioned and framed as “threatening, menacing, criminal, and in need of institutional control” (Leonard, 2010, p. 260). Christensen et al. (2016) theorize that public displays of Black athlete criminal VAW allows for the maintenance of the narrative that Black men are deviant, inherently violent, brutish, and in need of discipline. Media framing of alleged criminality by Black men also emphasizes individual choice and the behavioral problems of the person accused, rather than systemic racism or structural issues (Christensen et al., 2016; hooks, 2004a, 2004b).

## Method

### Arrested Player Sample

The sample of arrested players used in this study consists of 117 NFL players arrested for an alleged act of VAW between 2000 and 2019. This sample necessarily excludes NFL players who were accused in civil lawsuits, or those linked to incidents of VAW but never formally arrested, such as Ben Roethlisberger and Richie Incognito. It is possible that the lack of formal criminal charges laid against these players is reflective of certain race-based privileges (Chase, 2019). However, this is not within the scope of this study. This sample also excludes players accused of and even reprimanded for acts of VAW as youth or collegiate football players, like Joe Mixon or Tyreke Hill.

The 2000–2019 timeframe was chosen for several reasons. First, to provide a contemporary analysis of how arrests affect career outcomes, it is imperative to use data that is as recent as possible. However, the research questions in this study require a large-enough period of observing a player’s NFL career after the arrest to assess the career-level effects of that arrest. I tracked information on players arrested for alleged acts of VAW using Schroetenboer's (2021) *USA Today* NFL Arrests Database, which tracks all NFL player arrests from the year 2000 until the present, and has been used in past academic work (Leal et al., 2016, 2019; Rentner & Rentner, 2018).

I included in the sample players arrested for domestic violence, domestic assault, domestic battery, domestic abuse/assault, battery, or sexual assault of a woman. To ensure the accuracy of the *USA Today *Arrests Database, I corroborated the database by searching for the player’s name, their arrest or criminal charge (e.g., “domestic violence”), and the team they were playing for at the time of the arrest (e.g., “Atlanta Falcons”) on ProQuest’s North American journalism search engine. For each player, I found multiple news stories that corroborated the arrest information in the database and was able to fill in other key information regarding the relationship of the alleged victim to the athlete, the formal charges laid, and the conviction, sentence, or any disciplinary action doled out by the team or the NFL (if applicable). I then used data from pro-football-reference.com (Pro Football Statistics and History, 2020) to record the age of the player at the time of the arrest, their race, and their player statistics (described below).

### Matched Pairs Design

A matched-pairs research design is effective in assessing the impact that one intervening factor can have on an outcome, by comparing the results of an affected research subject with those of an unaffected, matched subject (Mallin et al., 1995). In this case, the intervening factor is an arrest for an act of VAW. To assess “before” and “after” data for matched, non-arrested players, the date (henceforth “intervention date”) for the matched player was determined based on the date of the arrest for the arrested player. For example, wide receiver Brandon Marshall was arrested on March 1, 2009, for domestic violence, after his third season in the NFL. The intervention date for both Marshall and the player matched with him, wide receiver Greg Jennings, is, therefore, March 1, 2009.

#### Criteria for Choosing Matched Pairs

Matched, non-arrested athletes were chosen based on similarities to arrested athletes on criteria that are most likely to determine NFL player longevity and salaries (Draisey, 2016; Mulholland & Jensen, 2019). These include age, draft round, player position, games played, games started, and on-field player value (described below) (Table 1).

**Table 1.** Summary Statistics for Matching Criteria—Arrested and Non-Arrested Player Sample.

Statistic	*N*	Mean	St. Dev.	Min	Max
*Arrested players*					
Age	117	25.974	2.920	21	35
Draft round	117	4.120	2.471	1	8
Before intervention seasons	117	3.839	2.708	0	12
Before intervention games played	117	50.316	40.080	0	180
Before intervention games started	117	30.974	34.570	0	180
Before intervention start percentage	117	0.493	0.349	0.000	1.000
Before intervention per-game AV	117	0.404	0.258	0.000	1.021
***Non-arrested players***					
Age	117	25.932	2.962	22	37
Draft round	117	4.111	2.525	1	8
Before intervention seasons	117	3.881	2.791	0	14
Before intervention games played	117	53.291	42.126	0	236
Before intervention games started	117	32.821	38.161	0	236
Before intervention start percentage	117	0.479	0.348	0.000	1.000
Before intervention per-game AV	117	0.389	0.247	0.000	1.000

I first matched players on their race and the position they played. Arrested players were labeled as either quarterback, running back, fullback, receiver, tight end, offensive lineman, defensive lineman, linebacker, cornerback, safety, and kicker. I then examined the players’ age, which had to be within two years of each other at the time of the arrest.

Next, I considered the players’ statistics. Due to the vastly different roles that players have in American football (i.e., running backs carry or catch the ball and record yards and touchdowns, while offensive linemen and defensive players almost never carry or catch the ball or record touchdowns), there are few statistics that could be systematically used across the sample to match players. I thus used games played, games started, approximate value per game, and participation in the Pro Bowl as matching criteria, due both to their capacity to predict salary (Draisey, 2016) and to their ubiquitous presence across all player positions. Approximate value is a statistic recorded by pro-football-reference.com that attempts “to put a single number on each player-season since 1960” (Pro Football Statistics, 2020). To standardize this statistic so as not to advantage players who have played longer than others, I calculated the approximate value on a per-game basis.

The following example illustrates the matching process: Ahman Green was arrested for domestic violence on April 25, 2005. I thus recorded Green’s race (Black), age at the time of arrest (27), position (running back), whether he participated in a Pro Bowl (he had), draft round (third), and player statistics for his seven seasons in the NFL preceding the arrest. In the seven seasons preceding the arrest, Green played in 107 games, starting 72. His per-game approximate value was 0.81, one of the highest recorded in the sample. His per-season approximate value was 12.4.

Using pro-football-reference.com’s player season finder tool, I then searched for a matched player based on as many of these parameters as possible, focusing the search on the seasons immediately preceding his arrest. For Green’s match, I searched for seasons played by Black running backs between the ages of 26 and 28, who played between the 2003 and 2004 NFL seasons, with an approximate value of 10 or higher. This initial search yielded seasons played by Shaun Alexander, Reuben Droughns, Edgerrin James, Fred Taylor, and Ricky Williams. While these players had seasons that matched the parameters described above, Alexander was the same age as Green (27) following the 2004 season, participated in a Pro Bowl, had started a similar percentage of games, and was closest in terms of his per-game approximate value. The original, self-created dataset with all 117 matched pairs (*n* = 234) is available as a supplemental file.

### Model

To measure the impact of VAW arrests on player career longevity, I use a hierarchical negative binomial model. This model predicts the length of a player’s career post-intervention, based on covariates for players at the individual level, and at the time level. The model, specified below, has two nested levels: players at the first level, and the calendar year at level 2. The dependent variable, denoted AY*it*, is the number of post-intervention seasons for player *i*, whose last pre-intervention season was at time *t.*

While post-intervention seasons do not directly account for player earnings from NFL salaries and endorsement and sponsorship contracts, it is a strong proxy for player career outcomes, as it measures how long a player remains employed by an NFL team. To measure post-intervention seasons, I recorded the number of seasons in which both arrested and matched players were paid a salary in the NFL *after* the intervention date (**AAfterYears)**. I perform a slight adjustment on the **(AfterYears)** variable to create the **(AAfterYears)** variable, adjusting for situations where players’ intervention date was in 2014 or later, and they played in 100% of their available post-intervention seasons. In these few cases, without adjusting, the amount of post-intervention years these players participated in would be right-censored relative to players from earlier years, because more recent players necessarily could only have played up to the 2019 season (even if they are still *currently* NFL players), compared to players arrested in years prior who had more potential seasons to play post-intervention. This adjustment only applied to 17 of the 234 players in the sample.

The primary explanatory variable is VAW arrests **(Arrests)**. This is recorded as 1 if the player was arrested for an act of VAW, and 0 if they were not. This arrest coefficient is impacted by second-level time variables, explained below. I also control for whether the player’s VAW arrest represents a repeat arrest (i.e., if they have been arrested for any other crime in the past).

This **Repeat** variable is coded as 1 if a player had been arrested once, 2 if they had been arrested twice, and 3 if they had been arrested three times in the past. There are also several other independent variables used to control for the impact of arrests on player outcomes at the individual-player level.

In the first level of the model, I control for the effect of player value, using both pre-intervention and post-intervention approximate values. As with the pre-intervention approximate value, I transform the post-intervention statistic so that it can be assessed on a per-game basis. Both approximate value statistics are standardized to have a mean of 0 and a one-unit standard deviation. I also include a covariate for how often players started games, both before and after the intervention. These “start percentage” statistics are both centered at their respective medians.

While it is generally common practice not to control for post-treatment variables, in this case it is vital to control for both pre-intervention and post-intervention player value when assessing the impact of arrests on a player’s career length. If post-intervention player value (measured by approximate value and start percentage) were not controlled for, a player’s career-ending shortly after their arrest could mistakenly be attributed to this arrest, rather than their lack of performance. Because the result of the outcome variable in question (career length) does not occur immediately after the arrest or intervention date, but rather over the rest of a player’s career, it is imperative that their performance and value over the rest of their career is controlled for. Arrests also do not directly impact a player’s performance, as arrested players had a mean start percentage (after-intervention) of 48.5% and a median start percentage of 54.6%, compared to 46% and 45.3%, respectively, for non-arrested players. Arrested players also had a mean approximate value per game of 0.26 (median 0.22), compared to 0.25 (median 0.24) for non-arrested players.

How long a player stays in the NFL following the intervention date also depends on how far into their career they were when the intervention occurred. This is measured by their pre-intervention seasons **(BeforeSeasons)**. Players who had only played 1 or 2 seasons before the intervention date could have many more years of NFL service ahead of them, while players whose intervention date is following their 8th or 9th season may have been already nearing retirement or the end of their careers. However, it is also possible that early in their careers, players are not yet established as stable performers in the NFL, and could be more likely to be out of the league soon. There could be a curvilinear relationship between pre-intervention seasons and post-intervention seasons. I, therefore, use both pre-intervention seasons **(BeforeSeasons_c)** and the square of pre-intervention seasons **(BeforeSeasons_c^2^)** as covariates in the first level of our model. **BeforeSeasons_c** is centered at its median of 3.

Where a player was drafted **(DraftRound)** also likely has an impact on their career length, even post-intervention. However, as a player's career advances and they get further away from their draft year, the importance of their draft position on their career longevity likely decreases. I, therefore, include an interaction effect between pre-intervention seasons and draft round, to account for this potential effect (DraftRound × BeforeSeasons). Draft round is recorded from 0 to 8 (though the draft only has seven rounds), with eight denoting undrafted players. I also control for the fixed effects of the race **(White)** and age **(Age_c)** of a player. White is coded as 1 if the player is white and 0 if not, while **Age_c** is centered to its median of 25.

The second level of the model examines the impact of the specific calendar year on the number of seasons a player plays post-intervention. This is done to assess whether the impact of an arrest on career length has changed over time, in line with perceived changing societal norms regarding VAW. The time level includes the intervention year, which denotes the last season a player played before intervention. This **Year** variable is standardized for the purpose of the analysis so that 1999 is recorded as 0, and 2019 (the last possible season) is recorded as 20. I also include a dichotomous variable for whether the arrest occurred before or after the Ray Rice assault and arrest **(Ray Rice)**. As explained above, this incident was a heavily covered media story, and in response, the NFL passed a new PCP, with specific clauses pertaining to acts of off-field violence. The effect of arrests **(Arrested)** on player seasons may also be impacted by the time at which they occur. The coefficient measuring the effect of arrests therefore is included at the second (time-based) level, with the same **Year** and **Ray Rice** variables. Including covariates at the second level for the effect of arrests will provide insight into the effect of arrests on career length, and how this effect has changed over time. While this does not ensure that the pre- and post-Ray Rice periods are of equal length, as in Spencer and Limperos’ (2018) study of media coverage, this strategy allows for an observation of year-over-year change as well.

The specifications of the model are shown below:

*AAfterYears_it_ ∼ Gam*(*λ_it_, θ*)

*(λ_it_) = α_#t_ + α_$t_Arrested_it_ + α_2_BeforeSeasons_it_ + α_&_BeforeSeasons^2^ + α_4_BeforeAV_it_*

+ _(_AfterAV_it_ + α_)_eStartPctC_it_ + α_*_AfterStartPctC_it_ + α_+_DraftRound

*∗ BeforeSeasons_it_ + α_9_AgeM_it_ + α_$#_White_it_ + α_$$_DraftRound_it_ + α_$2_Repeat_it_*

*α_#t_ = γ_##_ + γ_#$_InterventionYear + γ_#2_RayRice_t_ + η_#t_*

*α_$t_ = γ_$#_ + γ_$$_InterventionYear + γ_$2_RayRice_t_ + η_#t_*

## Results

The model fixed effect estimates are found in Table 2. The model parameters are estimated using the R package “brms,” with a Hamiltonian Monte Carlo sampler. Uninformative priors were used for parameter estimation.

**Table 2.** Multilevel Model Fixed Effects Estimates (Exponentiated).

	Estimate		Est. Error	95% credible interval		95% credible interval	
Intercept	1.096	(2.991)	0.185	0.730	1.465	(2.075)	(4.326)
Intervention year	−0.004	(0.996)	0.021	−0.045	0.037	(0.956)	(1.038)
Ray Rice	−0.352	(0.703)	0.281	−0.929	0.191	(0.395)	(1.211)
^a^Arrested	0.307	(1.359)	0.177	−0.029	0.653	(0.971)	(1.922)
Before intervention seasons^c^	−0.067	(0.935)	0.053	−0.169	0.035	(0.844)	(1.036)
Before intervention seasons squared^c^	0.002	(1.003)	0.005	−0.008	0.013	(0.992)	(1.013)
^b^Age^c^	−0.097	(0.907)	0.038	−0.173	−0.023	(0.841)	(0.978)
^a^Before intervention per game approximate value^d^	−0.094	(1.099)	0.071	−0.048	0.232	(0.954)	(1.261)
^b^After intervention approximate value^d^	0.263	(1.300)	0.084	0.098	0.428	(1.103)	(1.535)
Before intervention start percentage^c^	−0.266	(0.766)	0.206	−0.665	0.140	(0.514)	(1.150)
^b^After intervention start percentage^c^	0.877	(2.404)	0.229	0.423	1.328	(1.527)	(3.772)
Draft round	0.018	(1.018)	0.020	−0.021	0.056	(0.979)	(1.058)
Repeat	−0.024	(0.976)	0.083	−0.186	0.137	(0.830)	(1.147)
White	0.277	(1.319)	0.250	−0.239	0.762	(0.787)	(2.144)
Ray Rice: Arrested	0.119	(1.126)	0.333	−0.532	0.776	(0.587)	(2.173)
^a^Intervention year: Arrested	−0.039	(0.962)	0.024	−0.087	0.007	(0.917)	(1.007)
Before intervention seasons^c^: Draft round	0.004	(1.004)	0.008	−0.011	0.019	(0.989)	(1.019)

^a^ An effect within 90% credibility intervals (two-tailed).

^b^ An effect within 95% credibility intervals (two-tailed).

^c^ The variable was centered at its median.

^d^ The variable was standardized.

The baseline player in this analysis is a Black player of median age (25) who was a first-round draft pick, played the median number of seasons before the intervention year (3), and started a median percentage of his games both pre (52%) and post (49%) intervention. This baseline player’s last pre-intervention season was in 1999, with median per game approximate value statistics both pre (0.40) and post (0.24) intervention.^3^

Most of the fixed effects model estimates, for both the variables of interest and covariates, do not have a significant negative or positive effect on player career outcomes. The 95% credible intervals in Table 2 above indicate the probable range of estimates for the model, analogous to a confidence interval. However, it is important to note that these 95% credibility intervals are based on a two-tailed test of significance. Using a one-tailed test of significance, coefficient estimates of certain variables may still have a clear positive or negative impact on career length, as explained below.

Having been arrested for an act of VAW does not have a statistically significant positive or negative impact (within our two-tailed 95% credibility interval) on post-intervention career length. However, with a one-tailed test, the model produces 96.5% confidence that an arrest has a *positive* impact on a player's career. This confidence level is calculated using posterior predictive samples, which establish that 96.5% of the probability distribution for the “arrested” coefficient is above 0, indicating a positive effect. Without accounting for *when* the arrest happens, a baseline player (whose intervention year is in 1999) who has been arrested for an act of VAW is therefore likely to have played in approximately 35.9% *more* post-intervention seasons. Given that the baseline non-arrested player is expected to play in three seasons, an arrested counterpart is expected to play in 4.07 seasons, just over a one-season increase.

While an arrest may have a positive impact on a player's career without accounting for when it happens, the coefficient for *intervention year*: *arrested* shows that the effect of a VAW arrest on career length becomes more negative over time. As with the *arrested* coefficient, while a two-tailed test of significance does not yield 95% certainty that the effect of an arrest worsens over time, a one-tailed test produces 95.2% confidence that this effect is negative.

**Figure 1.**
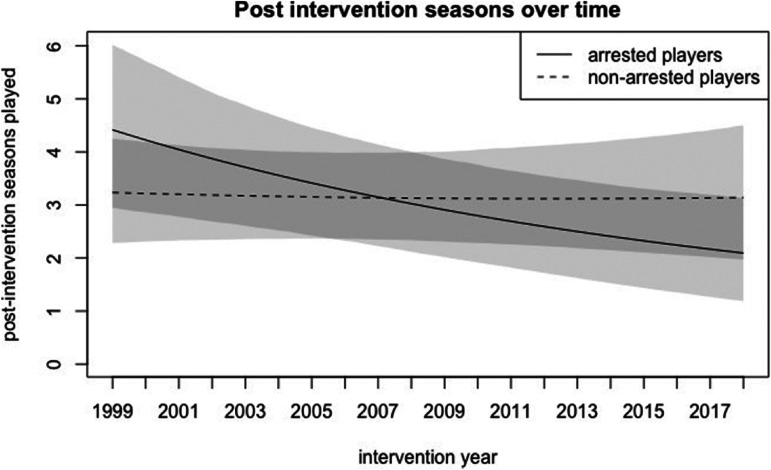
Impact of arrests on post-intervention seasons over time.

*Note.* Taking no other variable into account, arrested players are expected to play fewer post-intervention seasons than non-arrested players as of 2009. The shaded areas represent the error bars (at 95% confidence) for each group.

While players arrested in the early 2000s are predicted to play *more* post-intervention seasons than non-arrested players, over time, arrests have an increasingly negative impact on post-intervention seasons. This analysis indicates that the later (in the calendar year) a player is arrested, the more this arrest negatively impacts their career length. Using the mean estimate of the *intervention year: arrested* variable, an arrested player is expected to play 3.8% fewer post-intervention seasons for every year later that their arrest occurred. A baseline player arrested in 2006 therefore is expected to play 3.29 post-intervention seasons (compared to 4.07 for a player in 1999), while a baseline player arrested in 2015 is expected to play 2.34 post-intervention seasons. Up to and including players arrested in the year 2008, arrests are predicted to have either a slightly positive effect or no effect on a player's post-intervention seasons. For players arrested in 2009 and after, they are expected to participate in slightly fewer post-intervention seasons, with the negative impact of arrests increasing for every year following, as shown in Figure 1.

The impact of arrests on career outcomes was not clearly affected by whether the arrest occurred after the Ray Rice incident. Arrests that occurred after 2013 did have a more negative effect on the number of post-intervention seasons than arrests that happened in years prior, but this change was consistent with the year-over-year linear change described above. This analysis does not suggest that the effect of a year change between 2013 and 2014 (2013 being the last season before the Ray Rice incident occurred) was any different than the change between 2001 and 2002, 2007 and 2008, or any other 2 years in the sample.

Beyond the uncertain and changing (with time) impact of arrests, age has a clear negative impact on post-intervention seasons. For every year older a player was at intervention, they are expected to play 9% fewer seasons. Compared to a baseline 25-year-old player who is expected to play 3 seasons, a 29-year-old player is expected to play 2.1 seasons, a nearly one-season decrease.

**Figure 2.**
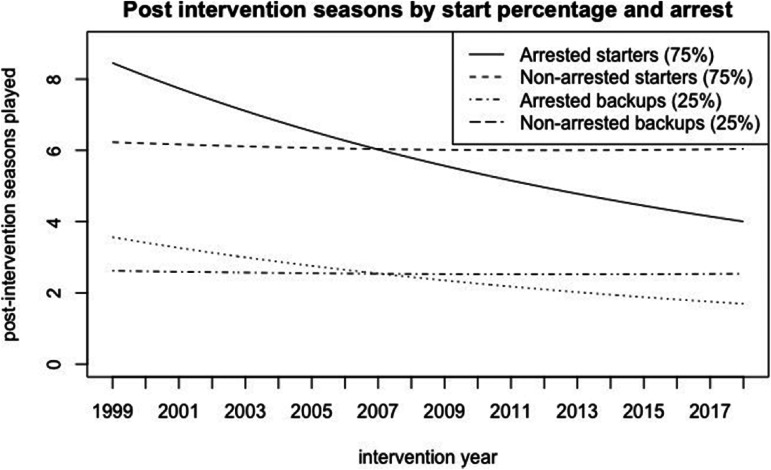
Impact of arrests and performance on post-intervention seasons overtime.

*Note.* Each bar represents a specific group of players, based on their start percentage and whether they were arrested. Arrested starters in the most recent year (2018) are still likely to play more post-intervention seasons than *non-arrested* backups at any time.

Player value and player performance also both have a positive effect on career length. Players who start a higher percentage of their games post-intervention are expected to play more post-intervention seasons, as the estimated coefficient for start percentage is 0.877, and its exponentiated coefficient is 2.40. To put this in context, this means that a baseline player who starts 25% of their games (a backup) is expected to play 1.95 seasons, while a player who starts 75% of their games (a starter) is expected to play 4.05 seasons. A baseline player who starts 0% of their games, who is in an exclusively backup role post-intervention, is only expected to play 0.9 seasons post-intervention. Even when the changing impact of an arrest over time is considered, an arrested starter in 2019 is expected to play more seasons than either an arrested or non-arrested backup in any year (Figure 2).

From a player performance perspective, for every one-unit standard deviation increase in post-intervention approximate value per game, a player is expected to play 30% more post-intervention seasons. Further, in analyses of subsamples where only the lowest-performing players—i.e., those who were more than 1 standard deviation below average in both approximate value and start percentage—were removed, the increasingly negative impact of arrests (based on the *Intervention Year: Arrested* coefficient) in later calendar years also disappears completely, even at lower credibility interval thresholds.^4^

Lastly, examining subsamples of only players who plead guilty or were convicted for their act of VAW (and matched pairs), both Bayesian and simple frequentist regression analyses show that arrested players do *not* play fewer seasons than their non-arrested counterparts. Arrests had no statistically significant impact on post-intervention seasons played, even in more recent years.

## Discussion

I assessed the popular claim that accusations and arrests for alleged acts of violence “ruin a person's career” (Levitt, 2013). The results from this Bayesian multi-level model point to negligible consequences for arrested athletes, especially higher performing players. Moreover, the model most conclusively demonstrates that on-field productivity and value have a much stronger and clearer impact on player careers. While it seems that the penalty for arrested athletes has increased over time, to the point that arrests for acts of VAW are predicted to have a small negative impact on career outcomes starting in the year 2009 (with this negative effect increasing in the following years), this change only affects lower-performing and lower-valued players.

While the impact of arrests seems to become increasingly negative over time for the full population of players, perhaps due to “much less tolerance for domestic violence than there was a decade ago” (Spencer & Limperos, 2018), a player's performance and value on the field seem to negate any negative impact that an arrest may have. Recent NFL history is littered with examples of higher-performing players who have been given second or third chances following incidents of off-field violence in both college and the NFL, including Tyreek Hill, Adrian Peterson, Antonio Brown, Brandon Marshall, and Ben Roethlisberger, among others (Freeman, 2021; M. Jacobs, 2021).

**Figure 3.**
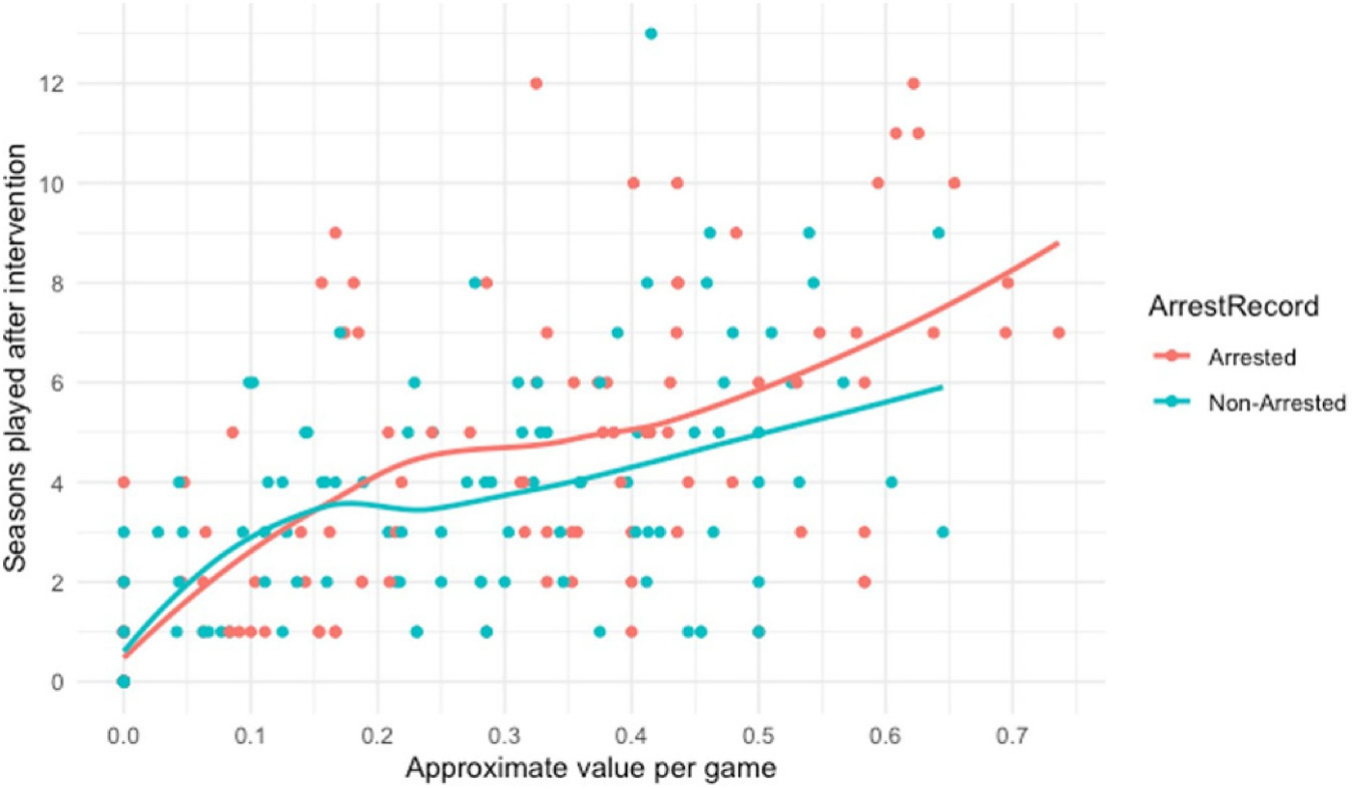
Post-intervention seasons played by approximate value, all players (*n* = 234).

*Note.* For both arrested and non-arrested players, performance (measured by approximate value) predicts post-intervention seasons played. This figure was made using loess smoothing for line fitting, and confidence interval error bars have been removed for clarity.

It is important to note that in this study, the “higher performance” subsamples analyzed did not include only top-performing players like Brown or Hill, but rather only excluded players in the bottom 25% of both start percentage and approximate value. Lower-performing players are much more likely to see their careers cut short, regardless of whether they have been arrested or not. This points to the possibility that NFL teams may be making examples of arrested lower-performing players by cutting them from their team or refusing to sign them to future contracts, to show that they are taking a stand against VAW. Jacobs (2021) makes this point with regard to the Seattle Seahawks’ January 2021 release of backup offensive lineman Chad Wheeler immediately following reports of a disturbing domestic violence incident. As Jacobs notes, “he's an easy one for the league to cast aside and use as an example of taking gender-based violence seriously” (2021), given that Wheeler's value to the team was not high to begin with (Figure 3).^5^

Cynics of sports and sports business may find these results unsurprising, as would some proponents of conflict perspectives, who could classify professional athletes as part of the wealthy, dominant class of people who are often not impacted by the law. While one should be cautious when generalizing the results beyond the group of NFL players analyzed here, this finding echoes the results of Sailofsky and Shor's analysis (2022) of NBA athletes, which shows that NBA players do not seem to be negatively impacted by arrests for acts of VAW if they are performing at even an adequate level. For most players, the results of this study suggest that the argument that accusations that do not result in conviction (or outright false accusations) are nearly or just as dangerous as acts of violence themselves is misplaced.

While I was not able to discuss how arrests may impact the public image and endorsement opportunities of players, for most players, their employment in the NFL is largely unaffected. Moreover, given the size of NFL rosters, the frequent turnover of players on teams and in the league more generally, and the NFL's general strategy of branding and promoting teams rather than individual players, most players have very little opportunity for sponsorship to begin with. The retention of players on teams and their continued presence in the league is a much more suitable indicator of career consequences.

For those who argue that players’ careers should indeed not suffer from arrests because the North American legal system operates under the premise that the accused is innocent until proven guilty, it is important to note that professional athletes are generally convicted at lower rates than those in the general population (Flood & Dyson, 2007). Moreover, as Robert Mueller (2015) noted in his independent investigation of the Ray Rice incident, there are “weaknesses inherent in the League's longstanding practice of deferring to the criminal justice system. … Discipline should be imposed on the basis of the specific nature of the player's conduct, not solely or necessarily on the disposition of a criminal case” (pp. 8-9). Of the 117 NFL players arrested for an act of VAW between 2000 and 2019, only 21 were found guilty. This 17.9% conviction rate is well below even the most conservative estimates of conviction rates for those arrested for domestic and sexual violence in the United States (Nelson, 2014). Of the 23 found guilty, four plead guilty to a lesser crime than they were initially accused of, and six served prison time. Moreover, arrests did not have a statistically significant negative impact on convicted or guilty players’ careers.

These general findings also stand in contrast with research on other employment contexts, which show that criminality has a significant negative effect on employment, particularly for Black men (Agan, 2017; Vuolo et al., 2017). Even though 111 of the 117 arrested NFL players in this study were Black, the number of seasons they played post-arrest was not substantially different than those who were not arrested. This highlights the effect that wealth, status, and perceived value have on how those arrested for acts of VAW are treated in both justice and employment contexts. For professional football players, it appears that these factors supersede the racial biases that typically impact Black men in criminal justice matters, at least in terms of the legal and extralegal sanctions suffered following arrests for acts of VAW.

According to the logic of deterrence and expressive theories of punishment, the lack of career punishment faced by NFL players accused of acts of violence against women could stunt efforts to reduce this violence, both at the professional and amateur levels. Studies from various non-sport contexts also suggest that the threat of future extralegal consequences and adverse effects in the labor market related to arrests may serve as a deterrent to future crime and violence (Bellair et al., 2017). Given the prevalence of off-field violence among collegiate football players (Beaver, 2019; McCray, 2019) and the status of NFL players as role models (Choi & Rifon, 2007), it is possible that the lack of punishment at the NFL level influences behavior at the collegiate level as well.

However, two notes of caution are important here. First, though my analysis indicates that the fear that allegations of VAW hurt player careers is empirically false for most NFL players, if this fear nevertheless exists, there could still be a deterrent effect among these players. To know whether NFL policies or team decision-making regarding violence has a deterrent effect on players’ likelihood to commit acts of VAW, one would need to assess whether players *believed* these arrests hurt their careers. Second, from a more practical perspective, one must also be cautious about how much power is granted to the NFL and its organizations to punish players without any sort of legal or investigatory process. As private organizations that are largely unaccountable to the public, there is a danger in providing these organizations with *carte blanche* to discipline players without due process, even in cases of alleged violence. This tension—between ensuring accountability for players’ bad behavior and ensuring that these players are treated fairly as workers by their powerful employers—is especially salient in the case of VAW by NFL players, as these situations usually involve both a group of alleged perpetrators who historically have been mistreated (young Black men) and a group of victims who historically have been ignored and underserved (women victims of men's violence).

It is also important to note that even changes in the extralegal sanctioning of NFL players are unlikely to eliminate off-the-field violence. There are many reasons for the continued prevalence of VAW perpetrated by athletes, and only some of them may be related to a lack of sufficient response and consequences. Professional sports, and professional football especially, is in many ways a breeding ground for fraternal, toxic masculinity that promotes violence and domination (Gage, 2008; Kreager, 2007). Starting at a young age, elite football players often receive preferential treatment from coaches, teachers, administrators, and parents. This can result in a belief that they are above the law, or that they will be protected from the consequences of any wrongdoing because of their talents (Trebon, 2007; Wallgren, 2009). Unfortunately, this belief may actually be warranted for NFL players, especially those performing at even an average level.

Another complicating factor in explaining how football might lead to violence is the risk of chronic traumatic encephalopathy (CTE) among players, due to repeated blows to the head and to concussions (Cummings et al., 2018). NFL players are particularly likely to suffer from CTE, which results in “a diverse set of clinical symptoms, including emotional and behavioral problems such as depression, anxiety, suicidality, aggression, and explosive bouts of anger” (Cummings et al., 2018, p. 710), which may manifest in violence. NFL players may also commit acts of VAW for some of the same reasons that those in the general population do, including growing up in poorer neighborhoods in precarious economic circumstances, in families where violence is normalized and learned, and in areas with less positive police presence (Johnson et al., 2007).

Nonetheless, social-control mechanisms do not seem to carry much weight in the NFL, which has implications for football players at all levels. While one must be cautious about generalizing the results of this study to other sports or employment contexts, which could have context-specific rules, policies, and cultures that affect how these arrests impact career outcomes, this analysis emphasizes the way elite performers are differentially impacted by legal and extralegal systems of control. As Jacobs (2021) writes, “if you’re exceptionally gifted at football, the NFL will almost always find a place for you no matter what you do in your spare time.”At the very least, an arrest for an act of VAW does not spell the end of an NFL player's career. In fact, when assessing his future in the league, an NFL player should be much more concerned with their performance on the field than their behavior off of it.

## Limitations

There are some limitations to this research that must be noted. As mentioned above, while the 117 arrested players represent the full population of NFL players arrested for an act of VAW over the past 20 years, this population remains relatively small. It is possible that this small sample explains the lack of statistical significance on some of the variable coefficients, and in the coefficients for some of the interaction terms. However, even with this relatively small sample, the models used still had good predictive power, and the interaction term for how the impact of an arrest changes over time was statistically significant.

It is also important to reiterate that career length only represents one important proxy for career outcomes. While this variable accounts for how long a player remains employed and earning a salary in the NFL, it does not account for the dollar amount of that salary, and whether they earn any other income through endorsement or sponsorship.

The qualitative analysis could also be used to examine why certain specific cases of VAW *do* impact athlete careers, as well as why other cases do not. Interviews of sport decision makers, as well as content analysis of media and social media, may be effective methodological tools for this type of analysis.

Finally, future research could examine how the #metoo movement has impacted prominent athletes accused of acts of VAW. While the most highly publicized stories from this movement have revolved around those in the entertainment industry, professional athletes are part of a similar group of famous, economically powerful men who are often protected from legal and extralegal consequences.

## Notes

1. As of September 17, 2021.

2. For the purposes of this article, “violence against women arrests” include arrests for domestic violence, domestic assault, domestic battery, domestic abuse, and battery, assault or sexual assault of a woman, regardless of whether the woman was a romantic partner of the player.

3. The pre-intervention approximate value statistic is substantially higher because of the inflated value given to a player's last pre-intervention season for matching purposes. This inflation was not done for the post-intervention approximate value statistics.

4. Subsample model results available upon request.

5. Figure 3 uses loess smoothing for line fitting, and confidence interval error bars have been removed for clarity.
